# Benzocaine and lidocaine induced methemoglobinemia after bronchoscopy: a case report

**DOI:** 10.1186/1752-1947-2-16

**Published:** 2008-01-23

**Authors:** Sophie Kwok, Jacqueline L Fischer, John D Rogers

**Affiliations:** 1 Department of Internal Medicine, University of Illinois College of Medicine at Peoria, Peoria, Illinois, USA

## Abstract

**Introduction:**

Methemoglobinemia is a rare cause of hypoxemia, characterized by abnormal levels of oxidized hemoglobin that cannot bind to and transport oxygen.

**Case presentation:**

A 62-year-old male underwent bronchoscopy where lidocaine oral solution and Hurricaine spray (20% benzocaine) were used. He developed central cyanosis and his oxygen saturation was 85% via pulse oximetry. An arterial blood gas revealed pH 7.45, P_CO2 _42, P_O2 _282, oxygen saturation 85%. Co-oximetry performed revealed a methemoglobin level of 17.5% (normal 0.6–2.5%). The patient was continued on 15 L/minute nonrebreathing face mask and subsequent oxygen saturation improved to 92% within two hours. With hemodynamic stability and improved SpO_2_, treatment with methylene blue was withheld.

**Conclusion:**

Methemoglobinemia is a potentially lethal condition after exposure to routinely used drugs. Physicians should be aware of this complication for early diagnosis and treatment.

## Introduction

Methemoglobinemia is an uncommon [1,2] but potentially fatal hemoglobinopathy. It leads to rapid oxygen desaturation, and therefore requires prompt recognition and treatment. This condition is often reported in the perioperative period when topical anesthetics are used during bronchoscopy, laryngoscopy, or upper gastrointestinal endoscopy. We present a case of a patient who developed methemoglobinemia after the use of both topical lidocaine and topical benzocaine for bronchoscopy.

## Case presentation

A 62-year-old Caucasian male with a past medical history of hypertension, hyperlipidemia, and cervical spine osteoarthritis was hospitalized for the problems of worsening chronic neck pain, new bilateral upper arm pain, and a persistent leukocytosis with an absolute monocytosis. His weight was 106.59 kg and height was 185.42 cm. His baseline hemoglobin and hematocrit were 11.1 grams/dL and 33.6% respectively. The described pain was intermittent, severe, and at times lancinating in nature. The patient underwent extensive diagnostic testing for the above mentioned problems and was ultimately diagnosed with complex regional pain syndrome. The patient was begun on steroid therapy and an improvement in his symptoms followed. The etiology of the leukocytosis and monocytosis remained unclear at the time of discharge. Other medications he received during hospitalization include: cephalexin, amitryptyline, amlodipine, enoxaparin, gabapentin, pantoprazole, oxycodone, and pravastatin.

During the hospitalization, a computed tomography (CT) scan of the chest, done as part of the investigation for the monocytosis, revealed bilateral ground glass pulmonary opacities. Further evaluation with bronchoscopy was performed. Topical pharyngeal anesthesia was achieved with 100 mL lidocaine hydrochloride solution orally, 4 mL of lidocaine aerosol, and 10 mL lidocaine jelly 2% topically. The patient was sedated with a total of 8 mg of midazolam and 50 mcg of fentanyl. His oropharynx was sprayed two times (one second each spray) with non-metered dose Hurricaine topical anesthetic aerosol spray (20% benzocaine) in preparation for bronchoscopy. The endoscope was inserted into the trachea and bronchi without difficulty.

During the procedure, the patient's oxygen saturation was 94% via pulse oximetry. Thirty minutes after the procedure, the patient developed central cyanosis, and his oxygen saturation decreased to 85% via pulse oximetry. His blood pressure was 152/77 mmHg and heart rate was 89 beats for minute. His cardiovascular and chest examinations were within normal limits. The patient did complain of being uncomfortable.

Oxygen was administered by nonrebreathing face mask initially at 10 L/minute, then at 15 L/minute when the cyanosis did not resolve. A chest radiograph was unremarkable. A chest CT was performed and showed no evidence of pulmonary embolism. An arterial blood gas revealed pH 7.45, P_CO2 _42, P_O2 _282, oxygen saturation 85%. The color of arterial blood was not noted. Co-oximetry performed revealed a methemoglobin level of 17.5% (normal 0.6–2.5%). A diagnosis of methemoglobinemia was made. The patient was continued on 15 L/minute nonrebreathing face mask and subsequent oxygen saturation improved to 92% within two hours. With hemodynamic stability and improved SpO_2_, treatment with methylene blue was withheld.

The patient's oxygen requirements lessened to 3 L/minute by nasal cannulae within 12 hours and his cyanosis resolved. Repeat arterial blood gas the next morning revealed a methemoglobin level of 1.1% by co-oximetry and the patient was doing well on room air. He had no adverse sequelae and the bronchoscopy revealed no abnormal findings. He remained in the hospital for several more days for treatment of his other medical conditions.

## Discussion

Methemoglobin develops when iron in hemoglobin is oxidized from the ferrous state (Fe^2+^) to the ferric state (Fe^3+^). When the iron of hemoglobin is oxidized to Fe^3+^, it is unable to carry oxygen. In healthy adults, methemoglobin accounts for less than 2% of total hemoglobin. This level is maintained primarily by the transfer of electrons from nicotinamide adenine dinucleotide (NADH) to NADH-cytochrome b5 reductase and then to cytochrome b5 [3].

Methemoglobinemia may be an inherited or acquired disorder. Inherited methemoglobinemia is rare and patients lack the enzyme NADH methemoglobin reductase (autosomal recessive deficiencies in cytochrome b5 or cytochrome b5 reductase). This form is most common in Alaskan Native Americans and individuals of Inuit descent [4,5]. Another less common form of congenital methemoglobinemia occurs in individuals who have an aberrant form of hemoblogin (HbM), where the reduced ferrous ion is destabilized and is more easily oxidized to a ferric ion. In addition, the enzyme methemoglobin reductase cannot interact with and efficiently reduce the methemoglobin in individuals who display this form of hemoglobin [6].

Acquired methemoglobinemia is more common than hereditary causes and occurs when an exogenous substance oxidizes hemoglobin producing methemoglobin at rates 100 to 1000 times greater than it can be metabolized. A wide variety of substances (Table 1) are known to induce methemoglobinemia, including amyl nitrite, nitroglycerin, dapsone, phenacetin, phenytoin, primaquine, sulfonamides, and local anesthetics such as lidocaine and benzocaine [7].

**Table 1:** Common medications and chemicals known to cause methemoglobinemia [7].

Acetaminophen	Hydroxylamine	Nitrosobenzene
Acetanilide	Local anesthetics	Phenacetin
Aminophenols	Benzocaine	Phenols
Ammonium nitrate	Cocaine	Phenytoin
Amyl nitrite	Lidocaine	Primaquine
Aniline	Prilocaine	Pyridine
Celecoxib	Procaine	Pyridium
Chloroquine	Menthol	Quinones
Cobalt	Methylene blue	Resorcinol
Chlorates	Nitrates/Nitrites	Silver nitrate
Copper sulfate	Nitrofuran	Sodium nitrite
Dapsone	Nitrogen oxide	Sulfonamides
EMLA	Nitroglycerin	Trinitrotoluene
Herbicides	Nitroprusside	

Factors that predispose to pharmacologic-induced methemoglobinemia include an excessive dose, a break in the normal mucosal barrier (which may increase the systemic absorption), and the concomitant use of other drugs known to cause methemoglobinemia.

Our patient received a combination of topical lidocaine and benzocaine, perhaps rendering him more susceptible to methemoglobinemia. A review of literature on lidocaine as a cause of methemoglobinemia is rarely reported. It almost always occurs in the setting of other agents and comorbidities [8]. Benzocaine is a more common cause of methemoglobinemia and reported more frequently in the literature. Based on one institution, the incidence of benzocaine-induced methemoglobinemia is one in 7000 bronchoscopies [1]; however, the exact incidence of methemoglobinemia associated with benzocaine is unknown. Because benzocaine is more lipophilic, it may continue to enter the blood stream from adipose tissue stores after methylene blue blood concentrations are no longer therapeutic [9]. Benzocaine is a more powerful oxidizing agent than lidocaine in animal studies, and a dose-response relationship has been demonstrated between benzocaine and methemoglobin [10,11]. Another risk factor for developing pharmacologic-induced methemoglobinemia is concomitant illnesses, such as cardiac and respiratory diseases [12]. Concentration of methemoglobin is reported as the percentage of total hemoglobin. Although hemoglobin level does not directly affect the production of methemoglobin, it does affect the amount of functional anemia. Our patient did have baseline anemia, which put him at a higher risk of developing more symptoms of methemoglobinemia. Furthermore, a non-metered dose Hurricaine topical anesthetic aerosol spray (20% benzocaine) was used in our patient, rather than a metered dose spray. The manufacturer recommends a dose of benzocaine 20% half-second spray that delivers 30 mg, so our patient probably received a relative overdose of benzocaine [13]. Infants are more susceptible than adults because hemoglobin F is more susceptible to oxidation [14]. In our case, the likelihood of an adverse drug reaction using the Naranjo probability scale was calculated to be probable (score of 6) [15]. Our conclusion was based on previous reports on this reaction; the adverse event appearing after the suspected drugs were administered; the adverse reaction improving when the drugs were discontinued; the drug being detected in the blood in a toxic concentration, and confirmation with objective evidence.

Clinical symptoms and signs depend on the level of methemoglobin. Levels greater than 15% are associated with cyanosis. Levels of 20–45% cause headache, anxiety, lethargy, tachycardia, lightheadedness, weakness, and dizziness. Dyspnea, acidosis, cardiac dysrhythmias, heart failure, seizures, and coma occur at levels above 45%. Methemoglobin levels above 60% are associated with a high mortality rate, and levels greater than 70% are fatal [3].

The diagnosis of methemoglobinemia is made by analysis of an arterial blood sample, using co-oximetry, which demonstrates a discrepancy between a low arterial oxyhemoglobin saturation (SaO_2_) and a relatively high arterial oxygen partial pressure (PaO_2_). A standard arterial blood gas analyzer measures the partial pressure of oxygen and calculates the oxygen saturation from this value. This is inaccurate because the methemoglobin level is assumed to be zero. However, a co-oximetry is a simplified spectophotometer that measure light absorbency at four different wavelengths and these wavelengths correspond to specific absorbency characteristics of deoxyhemoglobin, oxyhemoglobin, carboxyhemoglobin, and hemoglobin. In the presence of methemoglobinemia, oxygenation obtained by pulse oximetry is inaccurate because it does not reflect the degree of desaturation and can under or over estimate oxygenation depending on the severity of methemoglobinemia. The diagnosis should be suspected if cyanosis develops suddenly after the administration of oxidizing agents, or if chocolate brown arterial blood does not turn red on exposure to air [2].

In the absence of serious underlying illness, methemoglobin levels less than 30% usually resolve spontaneously over 15–20 hours when the offending agent is removed and oxygen is administered. Our patient did not receive methylene blue because he improved quickly with oxygen administration and his methemoglobin level was less than 30%. Methylene blue improves the efficiency of NADH methemoglobin reductase, and is an effective treatment for this condition. It is administered at a dose of 1–2 mg/kg IV slowly over 3–10 minutes. Improvement should occur within one hour, but if cyanosis persists, a second dose of methylene blue should be given [12]. Higher doses of methylene blue (> 7 mg/kg) may cause hemolysis and persistent cyanosis because the agent will oxidize hemoglobin to methemoglobin, instead of acting as a reducing agent at lower doses [13]. Methylene blue itself has side effects, which include nausea, vomiting, diarrhea, dyspnea, burning sensation in the mouth and abdomen, restlessness, and perspiration. The agent is an ineffective treatment for G6PD-deficient patients because G6PD generates NADPH, which acts as the reducing agent to convert methemoglobin to hemoglobin. Therefore, methylene blue would lead to the formation of more methemoglobin because of its oxidant potential, leading to hemolysis [3].

## Conclusion

Methemoglobinemia is a potentially severe complication of lidocaine and benzocaine, especially when used concomitantly. Among the acquired causes of methemoglobinemia, although caine-induced methemoglobinemia is rare, it may have a fatal outcome. Clinicians should, therefore, be familiar with this condition to ensure prompt diagnosis and effective treatment. Our patient responded promptly with supplemental oxygen and this case demonstrates that methylene blue is not always necessary in the treatment of methemoglobinemia.

## Competing interests

The authors declare that they have no competing interests.

## Authors' contributions

SK wrote and revised the manuscript. JLF and JDR reviewed and edited the paper. All authors approved the final manuscript.

## Consent

Consent for submission of this manuscript for publication has been given by the patient's wife.
